# Differential associations of somatic and cognitive-affective symptoms of depression with inflammation and insulin resistance: cross-sectional and longitudinal results from the Emotional Distress Sub-Study of the GRADE study

**DOI:** 10.1007/s00125-025-06369-8

**Published:** 2025-02-14

**Authors:** Dominic Ehrmann, Heidi Krause-Steinrauf, Diane Uschner, Hui Wen, Claire J. Hoogendoorn, Gladys Crespo-Ramos, Caroline Presley, Valerie L. Arends, Robert M. Cohen, W. Timothy Garvey, Thomas Martens, Holly J. Willis, Andrea Cherrington, Jeffrey S. Gonzalez, GRADE Research Group

**Affiliations:** 1 Research Institute Diabetes Academy Mergentheim (FIDAM), Bad Mergentheim, Germany; 2 Department of Clinical Psychology and Psychotherapy, University of Bamberg, Bamberg, Germany; 3 The Biostatistics Center, Department of Biostatistics and Bioinformatics, Milken Institute School of Public Health, The George Washington University, Rockville, MD, USA; 4 Ferkauf Graduate School of Psychology, Yeshiva University, Bronx, NY, USA; 5 Department of Medicine (Endocrinology), Albert Einstein College of Medicine, Bronx, NY, USA; 6 Department of Medicine (General Internal and Preventive Medicine), University of Alabama at Birmingham, Birmingham, AL, USA; 7 Advanced Research and Diagnostic Laboratory, Department of Laboratory Medicine and Pathology, University of Minnesota, Minneapolis, MN, USA; 8 Division of Endocrinology, Diabetes & Metabolism, Department of Medicine, University of Cincinnati College of Medicine & Endocrine Section, Cincinnati VA Medical Center, Cincinnati, OH, USA; 9 University of Alabama at Birmingham, Birmingham, AL, USA; 10 International Diabetes Center, HealthPartners Institute, Minneapolis, MN, USA; 11 Department of Epidemiology and Population Health, Albert Einstein College of Medicine, Bronx, NY, USA; 12 New York–Regional Center for Diabetes Translation Research, Albert Einstein College of Medicine, Bronx, NY, USA

**Keywords:** Depression, Inflammation, Insulin resistance, Somatic symptoms, Type 2 diabetes

## Abstract

**Aims/hypothesis:**

Insulin resistance and inflammation are components of a biological framework that is hypothesised to be shared by type 2 diabetes and depression. However, depressive symptoms include a large heterogeneity of somatic and cognitive-affective symptoms, and this may obscure the associations within this biological framework. Cross-sectional and longitudinal data were used to disentangle the contributions of insulin resistance and inflammation to somatic and cognitive-affective symptoms of depression.

**Methods:**

This secondary analysis used data from the Emotional Distress Sub-Study of the GRADE trial. Insulin resistance and inflammation were assessed using the HOMA-IR estimation and high-sensitivity C-reactive protein (hsCRP) levels, respectively, at baseline and at the study visits at year 1 and year 3 (HOMA-IR) and every 6 months (hsCRP) for up to 3 years of follow-up. Depressive symptoms were assessed at baseline using the Patient Health Questionnaire (PHQ-8), and a total score as well as symptom cluster scores for cognitive-affective and somatic symptoms were calculated. For the cross-sectional analyses, linear regression analyses were performed, with inflammation and insulin resistance at baseline as dependent variables. For the longitudinal analyses, linear mixed-effect regression analyses were performed, with inflammation and insulin resistance at the various time points as dependent variables. In all analyses, depressive symptoms (total score and symptom cluster scores) were the independent variables, controlled for important demographic, anthropometric and metabolic confounders. For the analysis of insulin resistance (HOMA-IR), data from 1321 participants were analysed. For the analysis of inflammation (hsCRP), data from 1739 participants were analysed.

**Results:**

In cross-sectional analysis and after adjustment for potential confounders, a one-unit increase in PHQ-8 total score was significantly associated with a 0.8% increase in HOMA-IR (*p*=0.007), but not with hsCRP (0.6% increase, *p*=0.283). The somatic symptom score was associated with a 5.8% increase in HOMA-IR (*p*=0.004). Single-item analyses of depressive symptoms showed that fatigue (3.6% increase, *p*=0.002) and increased/decreased appetite (3.5% increase, *p*=0.009) were significantly associated with HOMA-IR cross-sectionally. The cognitive-affective symptom score was not significantly associated with HOMA-IR at baseline. In longitudinal analyses, a one-unit increase in PHQ-8 total score was significantly associated with a 0.8% increase in hsCRP over time (*p*=0.014), but not with HOMA-IR over time (0.1% decrease, *p*=0.564). Again, only the somatic symptom cluster was significantly associated with hsCRP over time (5.2% increase, *p*=0.017), while the cognitive-affective symptom score was not.

**Conclusion/interpretation:**

The results highlight the associations of depressive symptoms with markers of inflammation and insulin resistance, both cross-sectionally and longitudinally, in individuals with type 2 diabetes. In particular, somatic symptoms of depression appear to be the driver of these associations, even after controlling for concomitant conditions, with a potential role for fatigue and issues with appetite.

**Trial registration:**

ClinicalTrials.gov NCT01794143

## Introduction

The prevalence of depression is nearly doubled in people with diabetes [1, 2]. This is a major clinical challenge, as depression not only negatively affects quality of life and glycaemic management [3, 4], but is also associated with an increased risk of microvascular and macrovascular complications, and higher mortality rates [5–7]. Previous research has suggested a bidirectional relationship between depression and type 2 diabetes, in which depression may be considered a risk factor for the development of type 2 diabetes, and, in turn, type 2 diabetes may be considered a risk factor for depression [8–10]. The same bidirectionality has also been discussed for the association between depression and diabetes complications [7].

This bidirectional relationship is not yet fully understood, and various behavioural (e.g. health behaviour), emotional (e.g. disease burden), social (e.g. socioeconomic status) and biological (e.g. systemic inflammation and insulin resistance) mechanisms may provide plausible explanations [4, 11]. A shared biological framework between depression and type 2 diabetes that involves systemic inflammation and the hypothalamic–pituitary–adrenal axis has been postulated [11].

Low-grade inflammation is consistently found in people with depression [12], people with type 2 diabetes [13] and people with type 2 diabetes and comorbid depression [14, 15]. Higher levels of high-sensitivity C-reactive protein (hsCRP) were also found to be associated with a higher risk for depression [16–18]. Previous research also showed associations between depression and insulin resistance [19, 20], even in people without type 2 diabetes [21].

Adding to the complexity in understanding the bidirectionality between depression and type 2 diabetes is the multifaceted nature of depression. Depression is a heterogeneous construct defined by the presence of self-reported symptoms that may be variously combined to satisfy the criteria for a diagnosis of major depressive disorder. Two individuals with the same diagnosis may share only one symptom [22, 23]. Thus, relying on total symptom scores may obscure potential differential effects of single symptoms or symptom clusters [24]. In diabetes, differential associations of somatic and cognitive-affective symptoms of depression have been demonstrated with regard to glycaemic management [25–27], self-management [28] and cardiovascular risk [29]. However, little is known about the differential associations of these symptom clusters with inflammation and insulin resistance in people with type 2 diabetes.

In this analysis, we aim to disentangle the differential associations of somatic and cognitive-affective depressive symptoms with insulin resistance and inflammation, and to analyse their role within a shared biological framework between depression and type 2 diabetes. In cross-sectional and longitudinal analyses, we analysed whether depressive symptoms are associated with parameters of inflammation and insulin resistance, and whether somatic or cognitive-affective symptoms play a key role in this association.

## Methods

For this secondary analysis, we used data from the Emotional Distress Sub-Study (EDS) of the Glycemia Reduction Approaches in Type 2 Diabetes: A Comparative Effectiveness (GRADE) study. The GRADE study was registered at ClinicalTrials.gov (registration number NCT01794143), and full details of the GRADE study and the EDS have been published previously [30, 31]. In brief, the GRADE study was an RCT that investigated the metabolic effects of four commonly used glucose-lowering medications (glargine [basal insulin U-100], glimepiride [a sulfonylurea], liraglutide [a glucagon-like peptide-1 agonist] and sitagliptin [a dipeptidyl peptidase-4 inhibitor]) in metformin-treated patients. The study enrolled 5047 participants from 36 clinical centres and nine additional subsites across the USA. The inclusion criteria for the GRADE study required individuals to be diagnosed with type 2 diabetes within the past 10 years, be aged 30 years or older (with the exception of American Indians and Alaska Natives who were eligible if they were 20 years old or older), have HbA_1c_ levels between 51 and 69 mmol/mol (6.8–8.5%), and be treated with metformin only. For the current secondary analysis, no additional inclusion criteria were applied. The GRADE RCT was designed to recruit and enrol a national sample that was broadly representative of the racial and ethnic diversity of individuals with type 2 diabetes in the USA. Clinical centres were selected for participation based on the availability of the target study population, and their ability to recruit a substantial proposition of racial/ethnic minorities (20% Black/African American and 18% Hispanic/Latino) as well as individuals aged 60 years and older (42%). The study assumed that there would be equal participation by men and women. The study collected data on participants’ self-reported sex during screening. Gender was not further ascertained. Overall, high representativeness can be assumed.

All GRADE sites were invited to participate in the EDS study; 26 of the 36 GRADE centres and eight subsites implemented the EDS protocol. The informed consent form used for the GRADE study was amended to include information about the EDS, and all local institutional review boards approved the protocol prior to implementation. Informed consent to participate in the EDS was obtained from all GRADE participants. A complete description of consent and participant enrolment is provided elsewhere [31]. The participating centres enrolled 1739 participants from 2015–2017, with the number of participants per centre ranging from 4 to 138. Participants received compensation for completing additional assessments, and were offered a copy of the ADA booklet ‘Diabetes and Your Emotional Health’ in English or Spanish. EDS participants completed the EDS assessments, consisting of a self-administered questionnaire battery and collection of a blood sample at baseline (before initiating the randomly assigned glucose-lowering medication) and at study visits every 6 months for up to 3 years of follow-up.

### Measures

Depressive symptoms were assessed using the Patient Health Questionnaire (PHQ-8) [32]. Total PHQ-8 scores range between 0 and 24, with a higher score indicating greater severity of depression symptoms. A score of ≥10 is indicative of clinically elevated levels of depressive symptoms. Cognitive-affective symptoms are assessed using three items: little interest or pleasure, feeling down, depressed or hopeless, and feeling bad about yourself. Somatic symptoms are assessed using five items: problems with sleep, fatigue, appetite, concentration and psychomotor slowing. For both sub-scores, the mean item score (range 0–3) was calculated to create a cognitive-affective and somatic symptom score, respectively [28].

Diabetes distress was assessed using the diabetes distress scale [33]. The diabetes distress scale reflects the overall intensity of diabetes distress, based on the mean responses (from 1–6) on 17 items. Diabetes distress was assessed because of its conceptual overlap with depressive symptoms [4].

Demographic and other participant characteristics (e.g. sex, age and race/ethnicity) were obtained through interviews conducted by research staff. All physical and metabolic measurements were performed by centrally trained certified staff [30, 31]. Medications for depression and anxiety were self-reported.

As measure of inflammation, hsCRP was assessed from blood samples collected at baseline and at visits every 6 months during follow-up (concurrent with the EDS questionnaire completion) for up to 36 months. The hsCRP values were log-transformed using the natural logarithm. Laboratory tests were performed by the Central Biochemistry Laboratory at the Advanced Research and Diagnostic Laboratory of the University of Minnesota. The hsCRP level was measured in serum on the Cobas c502 chemistry analyser (Roche Diagnostics) using a latex particle-enhanced immunoturbidimetric assay.

HOMA-IR was calculated as a measure of insulin resistance [34], and was derived from the HOMA2 index that uses glucose and C-peptide measurements obtained during the OGTT. It was calculated using the HOMA2 calculator version 2.2.3 (Diabetes Trials Unit, University of Oxford, Oxford, UK), and was assessed at baseline and at year 1 and 3 follow-up visits. HOMA-IR values were log-transformed using the natural logarithm.

### Statistical analysis

Analyses were conducted using all data for participants with non-missing data. For the inflammation analysis, of 1739 EDS participants, 1711 (98.4%) had complete baseline data and were included. For the insulin resistance analysis, of 1321 participants, 1294 (98.0%) had complete baseline data and were included in the analysis. Overall, there was a low level of missing data across time points (see electronic supplementary material [ESM] Fig. 1). Thus, no imputation approach was employed. The characteristics of the participants are described using mean ± SD for quantitative factors (e.g. age) and percentages for discrete factors (e.g. sex, income). For all analyses, the log_e_-transformed data for hsCRP and HOMA-IR, winsorised at the of 95% level, were used [35]. For the cross-sectional analyses, linear regression analyses were performed, with inflammation and insulin resistance at baseline as dependent variables. Independent variables of interest were either the PHQ-8 total score or the cognitive-affective or somatic symptom scores (analysed in separate analyses). All analyses were adjusted for diabetes distress, BMI, lipid profiles, whether the participant was taking lipid-lowering drugs or antidepressants, sex, age and race/ethnicity. For the longitudinal analyses, linear mixed-effect regression analyses were performed, with inflammation and insulin resistance over the various time points as dependent variables. Independent variables of interest were the baseline PHQ-8 total score, the baseline cognitive-affective symptom score or the baseline somatic symptom score (analysed in separate analyses). All longitudinal analyses were adjusted for baseline inflammation or baseline insulin resistance, diabetes distress at baseline, age, sex, race/ethnicity, BMI and HbA_1c_, lipid profiles, and whether the participant was taking lipid-lowering drugs or antidepressants. Additional covariates (randomised treatment group, baseline duration of type 2 diabetes) were added to the longitudinal analyses to reflect the changes in treatment and the course of diabetes from baseline. For each analysis, a random intercept was included for each participant, allowing each participant to have their own intercept and accounting for repeated measures over time as well as potential within-participant correlation. Covariates were selected based on previous literature [15, 36, 37].

Due to the effects of insulin therapy on HOMA-IR, the participants randomised to the insulin glargine group were excluded from all analyses relating to insulin resistance. Thus, the full-analysis set was used for analyses relating to inflammation, but a reduced analysis dataset was used for analyses relating to insulin resistance. For the longitudinal analyses on insulin resistance, sensitivity analyses were performed that excluded those participants who took insulin at any time point after being randomised to non-glargine treatment groups. In addition to HOMA-IR, the Matsuda index was also used to assess insulin resistance as a sensitivity analysis. The Matsuda index is calculated as a function of whole-body insulin sensitivity using fasting glucose and fasting insulin and mean glucose and mean insulin during an OGTT [38].

To investigate potential associations of HOMA-IR and hsCRP with depressive symptoms on a more granular level, further exploratory analyses were performed using separate regression models for each item of the PHQ-8 using the same statistical approaches as described above. However, these exploratory analyses were not pre-specified.

Given the exploratory nature of our analyses and to account for the potential increase in type I error associated with multiple comparisons, we used a tiered approach to statistical significance. For the main pre-specified analyses of cross-sectional and longitudinal associations of depressive symptoms and symptom clusters (cognitive-affective and somatic) with insulin resistance and inflammation, we used a significance level of 0.05; for the analyses that were not pre-specified (relating to single items of the PHQ-8), we set the significance level for type I error at 0.01. This threshold was selected to balance the risk of type I error with the need for statistical power to detect potentially meaningful effects across our primary outcome measures. Data were analysed centrally at the GRADE Coordinating Center at the George Washington University Biostatistics Center. The lme4 package from R version 4.2.1 (R Project for Statistical Computing) was used to fit the mixed-effect models.

### Ethics approval

GRADE is a multi-centre RCT, approved by over 30 institutional review boards. The primary review board submission information from the most recent annual renewal is: The George Washington University, Office of Human Research – Institutional Review Board; IRB number: 071245 (last approved: 6 August 2024; expires: 23 August 2025).

## Results

### Baseline characteristics

The baseline characteristics (Table 1) were comparable in the full EDS cohort (*N*=1739) and the reduced EDS cohort (*N*=1321). Participants had a mean age of 58.0 years (SD 10.2) and a diabetes duration of 4.2 years (SD 2.8). There were more male participants (67.6%) than female participants, and 56.1% were non-Hispanic White, 16.8% were Hispanic, 18.2% were non-Hispanic Black or African American and 8.9% self-identified as non-Hispanic other. Participants had a mean baseline HbA_1c_ of 58.6 mmol/mol (SD 5.3), mean BMI of 34.1 kg/m^2^, and approximately 41% had diabetes complications.

### Cross-sectional association of depressive symptoms with insulin resistance and inflammation

Table 2 presents the association of depressive symptoms (total scores and somatic and cognitive-affective symptom scores) at baseline with insulin resistance and inflammation at baseline, while controlling for diabetes distress, BMI, lipid profiles, whether the participant was taking lipid-lowering drugs or antidepressants, sex, age and race/ethnicity. Higher overall depressive symptoms (PHQ-8 total score) at baseline were significantly associated with higher baseline levels of HOMA-IR (*p*=0.007). Specifically, a one-unit increase in the PHQ-8 total score was associated with a 0.008 increase in the natural logarithm of insulin resistance, corresponding to a 0.8% increase on the original scale of insulin resistance. The baseline PHQ-8 total score was not significantly associated with baseline measures of hsCRP (*p*=0.283). Spline plots for the raw association between PHQ-8 scores and insulin resistance (ESM Figs 2 and 3) and inflammation (ESM Figs 4 and 5) indicate linear associations, thereby justifying the decision to include PHQ-8 scores as a continuous predictor.

Somatic symptom scores, but not cognitive-affective symptom scores, were significantly associated with HOMA-IR at baseline (*p*=0.004). A one-unit increase in the baseline somatic symptom score was associated with a 0.058 increase in the natural logarithm of baseline HOMA-IR, corresponding to a 5.8% increase on the original scale of insulin resistance (Table 2). There were no significant associations for either sub-score with hsCRP (*p*>0.05).

### Longitudinal association of depressive symptoms with insulin resistance and inflammation

Table 3 presents the associations of depressive symptoms at baseline (PHQ-8 total score, somatic and cognitive-affective symptom scores) with insulin resistance and inflammation over time, while controlling for the corresponding outcomes at baseline (insulin resistance/inflammation, respectively), baseline diabetes distress, baseline duration of type 2 diabetes, randomised treatment group and the above-mentioned covariates. The linear mixed-effect model showed no significant association of the PHQ-8 total score with HOMA-IR over time (*p*=0.564). For inflammation, there was a significant association between higher PHQ-8 total score at baseline with higher hsCRP over time (*p*=0.014). A one-unit increase in baseline PHQ-8 total score was associated with a 0.008 increase in the natural logarithm of inflammation over the 36-month follow-up period, corresponding to a 0.8% increase on the original scale.

Analyses of symptom clusters did not reveal any significant associations with HOMA-IR levels over time (*p*>0.05). However, for inflammation, somatic symptoms were significantly associated with hsCRP over time (*p*=0.017) (Table 3). A one-unit increase in the somatic symptom score at baseline was associated with a 5.2% increase in hsCRP over the 36-month follow-up. There was no association of a higher cognitive-affective symptom score at baseline with higher hsCRP over time (*p*=0.051).

### Sensitivity and exploratory analyses

The sensitivity analysis for insulin resistance that excluded participants who were taking insulin after randomisation (despite being randomised to the non-glargine groups; *n*=276/1321) yielded similar results. Neither the PHQ-8 total score nor the symptom scores were significantly associated with HOMA-IR levels over time (*p*>0.05; ESM Table 1). When using the Matsuda index instead of HOMA-IR, the results for the cross-sectional analysis revealed a non-significant association with PHQ-8 total and symptom scores (*p*>0.05; ESM Table 2). The longitudinal associations with the Matsuda index corroborated the results found for HOMA-IR (ESM Table 3).

Single-item analyses of the PHQ-8 revealed that the somatic symptoms ‘feeling tired or having little energy’ (estimate 0.036, *p*=0.002) and ‘poor appetite or overeating’ (estimate 0.035, *p*=0.009) in particular were significantly associated with insulin resistance at baseline (ESM Table 4). Longitudinally, ‘poor appetite or overeating’ (estimate 0.035, *p*= 0.015) was weakly but non-significantly associated with inflammation over time (ESM Table 5).

## Discussion

The current study set out to disentangle the differential associations of somatic and cognitive-affective symptoms with insulin resistance and inflammation within a shared biological framework between depression and type 2 diabetes. The results showed that total depressive symptoms were associated with higher insulin resistance at baseline and higher hsCRP during follow-up, and these associations were largely driven by somatic symptoms of depression. These findings provide further support for the shared biological framework underlying depression and type 2 diabetes, in that metabolic and immune dysregulation are associated with both diabetes and depressive symptom severity. This shared biological framework is further corroborated by the findings that the association was mainly driven by somatic symptoms, particularly fatigue/low energy and issues with appetite. Fatigue and hunger changes are central symptoms associated with immune dysregulation [39, 40], and these symptoms may also play a role in diabetes and glucose management.

The results from cross-sectional analyses corroborate previous findings of a bidirectional association between depression and incident type 2 diabetes [11, 21]. Thus, depression may play a role in elevated insulin resistance, although mechanistic studies are needed to analyse the directionality and causality of this association. Longitudinally, higher depressive symptom scores were associated with higher levels of inflammation over the 36-month follow-up period. The results also showed the relative importance of somatic symptoms over cognitive-affective symptoms. Therefore, in clinical practice, particular attention should be paid to typical somatic symptoms of depression, such as low energy or appetite problems, as these may both be considered risk factors for concomitantly higher levels of insulin resistance, and, in the long term, for higher levels of inflammation. However, cognitive-affective symptoms should also be addressed in clinical practice as they offer important information about potential emotional problems with diabetes.

The importance of somatic symptoms can also be seen in the strength of associations. A one-unit increase in somatic symptoms was associated with an approximately 5% increase in insulin resistance (cross-sectionally) and inflammation (longitudinally), compared with a relatively small increase (<1%) in these measures when analysing overall depressive symptoms. The clinical significance of these associations needs to be further evaluated. A better understanding of the causality is also needed, for example by analysing the effects of improving (somatic) depressive symptoms on the course of inflammation. Initial evidence for causality comes from Herder et al, who showed that, in people with diabetes, hsCRP levels showed significant associations with response to depression treatment, as reductions in depressive symptoms were associated with reductions in hsCRP level [36]. Furthermore, Zahn et al found that higher hsCRP levels attenuated the response to depression treatment [37].

The results also have some implications for diabetes complications. Inflammation plays an important role in the development of diabetes complications [41]. Thus, the longitudinal associations of overall depressive symptoms and somatic symptoms with inflammation are clinically relevant and may contribute to a better understanding of the association between depression and diabetes complications [7]. Although the observed effect size appear small, even modest increases in hsCRP may have clinical relevance over time. Previous research has shown that increases in hsCRP levels can significantly increase the risk of coronary heart disease [42, 43] and cancer [43]. Therefore, the observed longitudinal relationship between somatic symptoms of depression and hsCRP in people with type 2 diabetes may suggest potential long-term impacts on metabolic and cardiovascular health. However, further research is needed to fully understand the long-term effects and public health implications of these associations.

Our findings are in line with previous research demonstrating stronger associations of somatic symptoms with inflammation in people without diabetes [44, 45], and with research demonstrating the relative importance of somatic symptoms over cognitive-affective symptoms for people with diabetes [25, 26, 28, 29] and also for people with heart disease [46].

When considering somatic symptoms of depression in clinical practice, the wording of the PHQ-8 regarding sleep, appetite and psychomotor issues must be considered. Too much and too little sleep and appetite may be indicative of depression as well as psychomotor retardation and psychomotor agitation. This makes it difficult to infer any causal effects of these somatic symptoms on inflammation and insulin resistance. There is emerging evidence that increased and decreased appetite show differential associations with biomarkers of insulin resistance and inflammation. Simmons et al found that, while depressed individuals with decreased appetite showed higher cortisol levels, depressed individuals with increased appetite showed higher insulin resistance and inflammation levels (e.g. hsCRP) [47].

Of note, the associations between depressive symptoms and inflammation/insulin resistance were present after controlling for diabetes distress. Previous studies have shown that significant associations of depressive symptoms with glycaemic management vanished when the analyses controlled for diabetes distress [48, 49]. In this analysis, however, independent associations of overall depressive symptoms and the somatic symptom score were demonstrated. The current results highlight the importance of depressive symptoms, particularly somatic symptoms, as diabetes-unspecific factors that are associated with clinically important metabolic variables in people with a relatively short duration of type 2 diabetes (<10 years). This may also be seen as evidence that depression and diabetes distress are two independent constructs that share some conceptual overlap [4] but both show independent associations with relevant outcomes of diabetes management [3, 4]. Consequently, both should be monitored regularly in clinical practice, and standardised screening for both should be established [50].

When interpreting the findings, the following limitations must be considered. First, the presence of clinical depression or use of antidepressants was not assessed by a study clinician but rather by self-reporting. Second, given the large sample, the study had good power to detect small effects; however, the clinical significance of the observed effects between depressive symptoms and insulin resistance and inflammation remains unclear. In addition, the finding that insulin resistance only showed significant associations in the cross-sectional analysis while inflammation only showed significant associations in longitudinal analyses needs to be studied further. The existence of different mechanisms linking depressive symptoms to insulin resistance and inflammation may be responsible for these time-related differences. Furthermore, causality cannot be assumed and needs to be addressed in further studies. The difference in results for the cross-sectional associations when comparing the Matsuda index and HOMA-IR must be considered. However, due to missing data that are necessary to calculate the Matsuda index, we decided to use HOMA-IR for the main analyses. Only one biomarker of inflammation was assessed, but this further corroborated the importance of hsCRP [14]. However, further research should also focus on previously identified associations of depressive symptoms with IL-1RA, CCL2 (C-C motif chemokine ligand 2), adiponectin, IL-1β, IL-6 and IL-18 [14]. Finally, the levels of depressive symptoms were rather low in this clinical trial, affecting its representativeness and the ability to detect an effect due to limited variation. Furthermore, although women have been found to have higher prevalence of depression [1–3], more men participated, which may affect the generalisability of the study results despite controlling for the effect of sex in all analyses.

In summary, our results suggest that depressive symptoms are linked to insulin resistance and inflammation in the early stages of diabetes diagnosis, even in a sample with a relatively low prevalence of elevated depressive symptoms. While the clinical significance of the observed relationships among depressive symptoms and inflammation over time remains unclear, the results indicate the relative importance of somatic symptoms of depression over cognitive-affective symptoms. Our results contribute to a better understanding of the shared biological framework between depression and type 2 diabetes. In clinical practice, special attention should be given to somatic symptoms because of the identified associations with metabolic variables such as inflammation and insulin resistance, both cross-sectionally and longitudinally.

## Supplementary Material

Supplementary Material

## Figures and Tables

**Table 1:** Baseline characteristics of participants in the GRADE study EDS

Characteristic	Full EDS cohort (*N* = 1739)	Reduced EDS cohort^a^ (*N* = 1321)
Age (years)	58.0 ± 10.2	58.2 ± 10.3
Diabetes duration (years)	4.2 ± 2.8	4.2 ± 2.8
Gender		
Male	1175 (67.6)	887 (67.1)
Female	564 (32.4)	434 (32.9)
Race/ethnicity		
Hispanic	292 (16.8)	224 (17.0)
Non-Hispanic Black/African American	317 (18.2)	238 (18.0)
Non-Hispanic White	975 (56.1)	735 (55.6)
Non-Hispanic other^b^	155 (8.9)	124 (9.4)
Education		
College/graduate school	711 (40.9)	544 (41.2)
Some college	511 (29.4)	377 (28.5)
High school or less	517 (29.7)	400 (30.3)
Income (US dollars)^c^		
<10k	93 (6.2)	69 (6.1)
10–20k	163 (10.9)	116 (10.2)
20–50k	477 (31.8)	374 (33.0)
50k+	766 (51.1)	574 (50.7)
Living situation		
Alone	295 (17.0)	217 (16.4)
With another adult	1376 (79.1)	1052 (79.6)
With children only	68 (3.9)	52 (3.9)
Employment		
Currently employed full or part-time	974 (56.0)	742 (56.2)
Currently retired	459 (26.4)	349 (26.4)
Other	306 (17.6)	230 (17.4)
Smoking status		
Never	900 (51.8)	676 (51.2)
Past	617 (35.5)	474 (35.9)
Current	222 (12.8)	171 (12.9)
BMI (kg/m^2^)	34.1 ± 6.5	34.0 ± 6.5
Hypertension^d^	1486 (85.5)	1133 (85.8)
Diabetes complications^d^	716 (41.2)	550 (41.6)
Depression medications^d^	319 (18.3)	236 (17.9)
HbA_1c_ (mmol/mol)	58.6 ± 5.3	58.6 ± 5.3
HbA_1c_ (%)	7.5 ± 0.5	7.5 ± 0.5
log_e_ hsCRP	1.0 ± 1.0	1.0 ± 0.9
log_e_ HOMA-IR	−3.5 ± 0.4	−3.5 ± 0.4
PHQ-8 total score	3.4 ± 4.0	3.4 ± 4.0
Diabetes distress score	1.7 ± 0.7	1.7 ± 0.8
Somatic depression score	0.5 ± 0.5	0.5 ± 0.5
Cognitive-affective score	0.3 ± 0.5	0.3 ± 0.5

Data are reported as means ± SD for continuous variables or *n* (%) for categorical variables

a The reduced EDS cohort excludes participants who were randomised to receive glargine insulin

b For individuals categorised as non-Hispanic other, the racial groups/ethnicities reported include Hawaiian/Pacific Islander, other/multiple, Asian, American Indian/Alaska Native and unknown/not reported

c Income data were missing for 240 participants in the full cohort, leading to a denominator of 1499 for this variable; income data were missing for 188 participants in the reduced cohort, leading to a denominator of 1133

d Participants with hypertension or any diabetes complication, or taking any depression medication

**Table 2:** Cross-sectional associations of depressive symptoms (PHQ-8 total score, somatic and cognitive-affective symptom scores) with inflammation and insulin resistance at baseline

	Models	Independent variable	Inflammation (log_e_ hsCRP, *N* = 1739)				Insulin resistance (log_e_ HOMA-IR, *N* = 1321)			
			Estimate	SE	95% CI	*p* value	Estimate	SE	95% CI	*p* value
Overall depressive symptoms	Adjusted^a^	PHQ-8 total score	0.006	0.006	−0.005, 0.017	0.283	0.008	0.003	0.002, 0.013	0.007
	Unadjusted	PHQ-8 total score	0.034	0.006	0.022, 0.047	<0.001	0.020	0.003	0.013, 0.026	<0.001
Depressive symptom subscores	Adjusted^a^	Somatic	0.052	0.040	−0.026, 0.130	0.194	0.058	0.020	0.019, 0.097	0.004
		Cognitive-affective	0.017	0.041	−0.064, 0.098	0.676	0.036	0.021	−0.005, 0.077	0.088
	Unadjusted	Somatic	0.252	0.045	0.164, 0.340	<0.001	0.149	0.023	0.104, 0.193	<0.001
		Cognitive-affective	0.188	0.047	0.096, 0.280	<0.001	0.097	0.024	0.049, 0.144	<0.001

The insulin resistance models use the reduced EDS cohort, which excludes 418 participants randomised to glargine insulin. The unit of analysis is per one-unit increase in the PHQ-8 total score, somatic score or cognitive-affective score

a Adjusted for baseline age, sex, race/ethnicity, BMI, diabetes distress, lipid profiles and whether the participant was taking antidepressants or lipid-lowering medications

**Table 3:** Longitudinal associations of baseline depressive symptoms (PHQ-8 total score, somatic and cognitive-affective symptom scores) with inflammation and insulin resistance

	Models	Independent variable	Inflammation (log_e_ hsCRP, *N* = 1739)				Insulin resistance (log_e_ HOMA-IR, *N* = 1321)			
			Estimate	SE	95% CI	*p* value	Estimate	SE	95% CI	*p* value
Overall depressive symptoms	Adjusted^a^	PHQ-8 total score	0.008	0.003	0.002, 0.013	0.014	−0.001	0.001	−0.004, 0.002	0.564
	Unadjusted	PHQ-8 total score	0.039	0.006	0.027, 0.050	<0.001	0.016	0.003	0.011, 0.022	<0.001
Depressive symptom subscores	Adjusted^a^	Somatic	0.052	0.022	0.009, 0.095	0.017	−0.009	0.01	−0.029, 0.011	0.395
		Cognitive-affective	0.044	0.023	−0.0002, 0.089	0.051	0.001	0.011	−0.021, 0.022	0.963
	Unadjusted	Somatic	0.281	0.042	0.198, 0.365	<0.001	0.121	0.021	0.080, 0.162	<0.001
		Cognitive-affective	0.222	0.044	0.134, 0.309	<0.001	0.085	0.022	0.041, 0.129	<0.001

Models accounted for the within-participant correlation over time using a random effect

Insulin resistance models use the reduced EDS cohort, which excludes 418 participants randomised to glargine insulin. The unit of analysis is per one-unit increase in the PHQ-8 total score, somatic score or cognitive-affective score

a Adjusted for sex, race/ethnicity, randomised treatment group, and baseline measurements of age, duration of diabetes, HbA_1c_, BMI, lipid profiles, whether the participant was taking antidepressants or lipid-lowering medications, levels of inflammation and insulin resistance, and diabetes distress

## Data Availability

This manuscript is based on follow-up data and outcome assessments from the 1739 participants enrolled into the Emotional Distress Sub-Study (EDS) of the Glycemia Reduction Approaches in Type 2 Diabetes: A Comparative Effectiveness (GRADE) study. The GRADE and EDS database will be available in 2025 at the NIDDK Data Repository (https://repository.niddk.nih.gov/study/151).

## Research in context

### What is already known about this subject?

- Insulin resistance and inflammation are potential shared biological frameworks linking depression and type 2 diabetes
- Depression is a heterogeneous construct that may present with cognitive-affective symptoms as well as somatic symptoms
- Cross-sectional and longitudinal associations of these symptom clusters with insulin resistance and inflammation in people with diabetes have not been reported

### What is the key question?

- How are the various symptom clusters and symptoms of depression associated with markers of insulin resistance and inflammation at baseline and over time?

### What are the new findings?

- Overall, depressive symptoms are associated with more severe insulin resistance cross-sectionally, and with increased inflammation levels over time
- Somatic symptoms, rather than cognitive-affective symptoms, were the drivers of both of these associations
- There may be a particular role for fatigue and issues with appetite as single depressive symptoms

### How might this impact on clinical practice in the foreseeable future?

- The results contribute to a better understanding of a shared biological framework between depression and type 2 diabetes, by disentangling the differential associations of various depressive symptoms and highlighting the role of somatic symptoms

## Abbreviations

EDS: Emotional Distress Sub-Study
GRADE: Glycemia Reduction Approaches in Type 2 Diabetes: A Comparative Effectiveness study
hsCRP: High-sensitivity C-reactive protein
PHQ-8: Patient Health Questionnaire
