# Correction: Differential associations of somatic and cognitive-affective symptoms of depression with inflammation and insulin resistance: cross-sectional and longitudinal results from the Emotional Distress Sub-Study of the GRADE study

**DOI:** 10.1007/s00125-025-06433-3

**Published:** 2025-04-22

**Authors:** Dominic Ehrmann, Heidi Krause-Steinrauf, Diane Uschner, Hui Wen, Claire J. Hoogendoorn, Gladys Crespo-Ramos, Caroline Presley, Valerie L. Arends, Robert M. Cohen, W. Timothy Garvey, Thomas Martens, Holly J. Willis, Andrea Cherrington, Jeffrey S. Gonzalez, GRADE Research Group, J. P. Crandall, M. D. McKee, S. Behringer-Massera, J. Brown-Friday, E. Xhori, K. Ballentine-Cargill, S. Duran, H. Estrella, S. Gonzalez de la Torre, J. Lukin, L. S. Phillips, E. Burgess, D. Olson, M. Rhee, P. Wilson, T. S. Raines, J. Boers, J. Costello, M. Maher-Albertelli, R. Mungara, L. Savoye, C. A. White, C. Gullett, L. Holloway, F. Morehead, S. Person, M. Sibymon, S. Tanukonda, C. Adams, A. Ross, A. Balasubramanyam, R. Gaba, E. Gonzalez Hattery, A. Ideozu, J. Jimenez, G. Montes, C. Wright, F. Ismail-Beigi, C. Falck-Ytter, L. Sayyed Kassem, A. Sood, M. Tiktin, T. Kulow, C. Newman, K. A. Stancil, B. Cramer, J. Iacoboni, M. V. Kononets, C. Sanders, L. Tucker, A. Werner, A. Maxwell, G. McPhee, C. Patel, L. Colosimo, A. Krol, R. Goland, J. Pring, L. Alfano, P. Kringas, C. Hausheer, J. Tejada, K. Gumpel, A. Kirpitch, H. Schneier, C. N. Mariash, K. J. Mather, H. M. Ismail, A. Lteif, M. Mullen, T. Hamilton, N. Patel, G. Riera, M. Jackson, V. Pirics, D. Aguillar, D. Howard, S. Hurt, R. Bergenstal, A. Carlson, M. Johnson, R. Hill, J. Hyatt, C. Jensen, M. Madden, D. Martin, W. Konerza, S. Yang, K. Kleeberger, R. Passi, S. Fortmann, M. Herson, K. Mularski, H. Glauber, J. Prihoda, B. Ash, C. Carlson, P. A. Ramey, E. Schield, B. Torgrimson-Ojerio, K. Arnold, B. Kauffman, E. Panos, S. Sahnow, K. Bays, K. Berame, J. Cook, D. Ghioni, J. Gluth, K. Schell, J. Criscola, C. Friason, S. Jones, S. Nazarov, D. Wexler, M. E. Larkin, J. Meigs, B. Chambers, A. Dushkin, G. Rocchio, M. Yepes, B. Steiner, H. Dulin, M. Cayford, K. Chu, A. DeManbey, M. Hillard, K. Martin, N. Thangthaeng, L. Gurry, R. Kochis, E. Raymond, V. Ripley, C. Stevens, J. Park, V. Aroda, A. Ghazi, M. Magee, Ann Ressing, A. Loveland, M. Hamm, M. Hurtado, A. Kuhn, J. Leger, L. Manandhar, F. Mwicigi, O. Sanchez, T. Young, R. Garg, V. Lagari-Libhaber, H. J. Florez, W. M. Valencia, J. Marks, S. Casula, L. Oropesa-Gonzalez, L. Hue, A. Cuadot, R. Nieto-Martinez, A. K. Riccio Veliz, M. Gutt, Y. J. Kendal, B. Veciana, S. H. Hox, H. Petrovitch, M. Matwichyna, V. Jenkins, L. Broadwater, R. R. Ishii, N. O. Bermudez, D. S. Hsia, W. T. Cefalu, F. L. Greenway, C. Waguespack, E. King, G. Fry, A. Dragg, B. Gildersleeve, J. Arceneaux, N. Haynes, A. Thomassie, M. Pavlionis, B. Bourgeois, C. Hazlett, J. Krakoff, J. M. Curtis, T. Killean, M. Khalid, E. Joshevama, E. Diaz, K. Tsingine, T. Karshner, M. A. Banerji, P. August, M. Lee, D. Lorber, N. M. Brown, D. H. Josephson, L. L. Thomas, M. Tsovian, A. Cherian, M. H. Jacobson, M. M. Mishko, D. Dyer, M. C. R. Lawson, O. Griffith, A. Agne, S. McCullars, J. Craig, M. C. Rogge, K. Burton, K. Kersey, C. Wilson, S. Lipp, M. B. Vonder Meulen, C. Adkins, T. Onadeko, N. Rasouli, C. Baker, E. Schroeder, M. Razzaghi, C. Lyon, R. Penaloza, C. Underkofler, R. Lorch, S. Douglass, S. Steiner, W. I. Sivitz, E. Cline, L. K. Knosp, J. McConnell, T. Lowe, W. H. Herman, R. Pop-Busui, M. H. Tan, C. Martin, A. Waltje, A. Katona, L. Goodhall, R. Eggleston, S. Kuo, S. Bojescu, S. Bule, N. Kessler, E. LaSalle, K. Whitley, E. R. Seaquist, A. Bantle, T. Harindhanavudhi, A. Kumar, B. Redmon, J. Bantle, M. Coe, M. Mech, A. Taddese, L. Lesne, S. Smith, C. Desouza, L. Kuechenmeister, V. Shivaswamy, S. Burbach, M. G. Rodriguez, K. Seipel, A. Alfred, A. L. Morales, J. Eggert, G. Lord, W. Taylor, R. Tillson, D. S. Schade, A. Adolphe, M. Burge, E. Duran-Valdez, J. Martinez, A. Bancroft, S. Kunkel, F. Ali Jamaleddin Ahmad, D. Hernandez McGinnis, B. Pucchetti, E. Scripsick, A. Zamorano, P. Raskin, C. Rhee, S. Abraham, L. F. Jordan, S. Sao, L. Morton, O. Smith, L. Osornio Walker, L. Schnurr-Breen, R. Ayala, R. B. Kreymer, D. Sturgess, K. M. Utzschneider, S. E. Kahn, L. Alarcon-Casas Wright, E. J. Boyko, E. C. Tsai, D. L. Trence, S. Trikudanathan, B. N. Fattaleh, B. K. Montgomery, K. M. Atkinson, A. Kozedub, T. Concepcion, C. Moak, N. Prikhodko, S. Rhothisen, W. Tamborlane, A. Camp, B. Gulanski, S. E. Inzucchi, K. Pham, M. Alguard, P. Gatcomb, K. Lessard, M. Perez, L. Iannone, E. Magenheimer, A. Montosa, J. Fradkin, H. B. Burch, A. A. Bremer, D. M. Nathan, J. M. Lachin, J. B. Buse, N. Younes, I. Bebu, N. Butera, C. J. Buys, A. Fagan, Y. Gao, A. Ghosh, M. R. Gramzinski, S. D. Hall, E. Kazemi, E. Legowski, H. Liu, C. Suratt, M. Tripputi, A. Arey, M. Backman, J. Bethepu, C. Lund, P. Mangat Dhaliwal, P. McGee, E. Mesimer, L. Ngo, M. Steffes, J. Seegmiller, A. Saenger, D. Gabrielson, T. Conner, S. Warren, J. Day, J. Huminik, A. Scrymgeour, Ivan Abdouch, Gul Bahtiyar, Paula Brantley, Frances E. Broyles, Gay Canaris, Paul Copeland, Jeri J. Craine, Warren L. Fein, Agnieska Gliwa, Lisel M. Hope, Melissa S. Lee, Rebecca Meiners, Vaughan Meiners, Hollis O’Neal, James E. Park, Alan Sacerdote, Edward Sledge, Lisa Soni, Jeanne Steppel-Reznik, Alexander Turchin, Sherita H. Golden, Aanand D. Naik, Elizabeth A. Walker

**Affiliations:** 1 Research Institute Diabetes Academy Mergentheim (FIDAM), Bad Mergentheim, Germany; 2 Department of Clinical Psychology and Psychotherapy, University of Bamberg, Bamberg, Germany; 3 The Biostatistics Center, Department of Biostatistics and Bioinformatics, Milken Institute School of Public Health, the George Washington University, Rockville, MD USA; 4 Ferkauf Graduate School of Psychology, Yeshiva University, Bronx, NY USA; 5 Department of Medicine (Endocrinology), Albert Einstein College of Medicine, Bronx, NY USA; 6 Department of Medicine (General Internal and Preventive Medicine), University of Alabama at Birmingham, Birmingham, AL USA; 7 Advanced Research and Diagnostic Laboratory, Department of Laboratory Medicine and Pathology, University of Minnesota, Minneapolis, MN USA; 8 Division of Endocrinology, Diabetes & Metabolism, Department of Medicine, University of Cincinnati College of Medicine & Endocrine Section, Cincinnati VA Medical Center, Cincinnati, OH USA; 9 University of Alabama at Birmingham, Birmingham, AL USA; 10 International Diabetes Center, HealthPartners Institute, Minneapolis, MN USA; 11 Department of Epidemiology and Population Health, Albert Einstein College of Medicine, Bronx, NY USA; 12 New York–Regional Center for Diabetes Translation Research, Albert Einstein College of Medicine, Bronx, NY USA

**Correction: Diabetologia**

10.1007/s00125-025-06369-8

Some GRADE Research Group investigators were not identified as collaborators in the Acknowledgements. The original article has been corrected.

