# Supplementary material for: Differential associations of somatic and cognitive-affective symptoms of depression with inflammation and insulin resistance: cross-sectional and longitudinal results from the Emotional Distress Sub-Study of the GRADE study
Source: Diabetologia. 2025 Feb 14;68(7):1403–15. doi: 10.1007/s00125-025-06369-8 (PMC12176517; doi:10.1007/s00125-025-06369-8)
Supplement: Supplementary file 1 — ESM (PDF 800 KB) [file 125_2025_6369_MOESM1_ESM.pdf]

Supplementary Appendix

Differential associations of somatic and cognitive-affective symptoms of depression with inflammation and insulin resistance: cross-sectional and longitudinal results from the Emotional Distress Sub-Study of GRADE study

Dominic Ehrmann, Heidi Krause-Steinrauf, Diane Uschner, Hui Wen, Claire J. Hoogendoorn, Gladys Crespo-Ramos, Caroline Presley, Valerie Arends, Robert M. Cohen, W. Timothy Garvey, Thomas Martens, Holly Willis, Andrea Cherrington, Jeffrey S. Gonzalez, and the GRADE Research Group

Table of Contents

ESM Appendix 1. GRADE Research Group (April 30, 2021) 2

ESM Figure 1. Overview of missing values for the different variables included in the analyses. 8

ESM Figure 2. Relationship between PHQ-8 total score and insulin resistance ($\log_e$ HOMA-IR) at baseline. 9

ESM Figure 3. Relationship between PHQ-8 total score and insulin resistance ($\log_e$ HOMA-IR) at baseline with Locally Estimated Scatterplot Smoothing (LOESS) 10

ESM Figure 4. Relationship between PHQ-8 total score and inflammation ($\log_e$ hsCRP) at baseline. 11

ESM Figure 5. Relationship between PHQ-8 total score and inflammation ($\log_e$ hsCRP) at baseline with Locally Estimated Scatterplot Smoothing (LOESS) 12

ESM Table 1. Sensitivity analysis for the associations with insulin resistance excluding those taking insulin after randomisation 13

ESM Table 2. Sensitivity analysis for the cross-sectional associations with insulin resistance (comparing Matsuda Index & HOMA-IR) 14

ESM Table 3. Sensitivity analysis for the longitudinal associations with insulin resistance (comparing Matsuda & HOMA-IR) 15

ESM Table 4. Association between each depression symptom at baseline with inflammation and insulin resistance at baseline 16

ESM Table 5. Associations between each depression symptom at baseline and longitudinal inflammation and insulin resistance 18

ESM Appendix 1. GRADE Research Group (April 30, 2021)
Designations: Principal Investigator (PI); Co-Principal Investigator (Co-PI); Co-Investigator (Co-I); Study Coordinator (SC); Recruitment/Retention Coordinator (RC); Research Staff (RS)

Current Clinical Centres

Albert Einstein College of Medicine: Crandall, JP (PI); McKee, MD (Co-PI, past); Behringer-Massera , S (Co-I, past); Brown-Friday, J (SC); Xhori, E (RC, past); Ballentine-Cargill, K (RS); Duran, S (RS); Estrella, H (RS); Gonzalez de la torre, S (RS, past); Lukin, J (RS, past)

Atlanta VA Medical Centre: Phillips, LS (PI); Burgess, E (Co-I); Olson, D (Co-I); Rhee, M (Co-I); Wilson, P (Co-I); Raines, TS (SC); Boers, J (SC); Costello, J (SC); Maher-Albertelli, M (SC); Mungara, R (SC); Savoye, L (SC, past); White, CA (SC); Gullett, C (SC; past); Holloway, L (SC, past); Morehead, F (SC, past); Person, S (SC, past); Sibymon, M (SC, past); Tanukonda, S (SC, past); Adams, C (RC, past); Ross, A (RC, past)

Baylor Scott & White Research Institute (Baylor Research Institute): Balasubramanyam, A (PI); Gaba, R (Co-I); Gonzalez Hattery, E (SC); Ideozu, A (SC); Jimenez, J (SC); Montes, G (RC); Wright, C (RS, past)

Baylor Research Institute: Hollander, P (PI); Roe, E (Co-I, past); Jackson, A (SC); Smiley, A (SC); Burt, P (SC, past); Estrada, L (RS); Chionh, K (RS, past)

Case Western Reserve University/Cleveland VA/MetroHealth Medical Centre: Ismail-Beigi, F (PI); Falck-Ytter, C (Co-PI); Sayyed Kassem, L (Co-PI); Sood, A (Co-PI, past); Tiktin, M (Co-I, SC); Kulow, T (SC); Newman, C (SC); Stancil, KA (SC); Cramer, B (SC, past); Iacoboni, J (SC, past); Kononets, MV (SC, past); Sanders, C (SC, past); Tucker, L (SC, past); Werner, A (SC, past); Maxwell, A (RS); McPhee, G (RS); Patel, C (RS); Colosimo, L (RS, past); Krol, A (RS, past)

Columbia University Medical Centre: Goland, R (PI); Pring, J (SC); Alfano, L (SC); Kringas, P (SC, past); Hausheer, C (RC, past); Tejada, J (RC, past); Gumpel, K (RS, past); Kirpitch, A (RS, past); Schneier, H (RS, past)

Duke University Medical Centre: Green, JB (PI); AbouAssi, H (Co-I); Chatterjee, R (Co-I); Feinglos, MN (Co-I, past); English Jones, J (SC, RC); Khan, SA (SC, RC); Kimpel, JB (SC, past); Zimmer, RP (SC, past); Furst, M (RC, past); Satterwhite, BM (RS); Thacker, CR (RS); Evans Kreider, K (RS, past)

Indiana University: Mariash, CN (PI); Mather, KJ (PI, past); Ismail, HM (Co-I); Lteif, A (Co-I, past); Mullen, M (SC); Hamilton, T (SC, past); Patel, N (SC, past); Riera, G (RC); Jackson, M (RC, past); Pirics, V (RC, past); Aguillar, D (RS, past); Howard, D (RS, past); Hurt, S (RS, past)

International Diabetes Centre: Bergenstal, R (PI); Carlson, A (Co-I); Martens, T (Co-I); Johnson, M (SC); Hill, R (SC); Hyatt, J (SC); Jensen, C (SC); Madden, M (SC); Martin, D (SC); Willis, H (SC); Konerza, W (RS); Yang, S (RS); Kleeberger, K (RS, past); Passi, R (RS, past)

Kaiser Permanente Northwest: Fortmann, S (PI); Herson, M (Co-I); Mularski, K (Co-I); Glauber, H (Co-I, past); Prihoda, J (Co-I, past); Ash, B (SC); Carlson, C (SC); Ramey, PA (SC); Schield, E (SC); Torgrimson-Ojerio, B (SC); Arnold, K (SC, past); Kauffman, B (SC, past); Panos, E (SC, past); Sahnow, S

(RC); Bays, K (RS); Berame, K (RS); Cook, J (RS); Ghioni, D (RS); Gluth, J (RS); Schell, K (RS); Criscola, J (RS, past); Friason, C (RS, past); Jones, S (RS, past); Nazarov, S (RS, past)

Kaiser Permanente of Georgia: Barzilay, J (PI); Rassouli, N (Co-PI); Puttnam, R (Co-I); Ojoawo, B (SC); Nelson, R (RC); Curtis, M (SC, past); Hollis, B (SC, past); Sanders-Jones, C (SC, past); Stokes, K (SC, past); El-Haqq, Z (RS, past); Kolli, A (RS, past); Tran, T (RS, past)

Massachusetts General Hospital: Wexler, D (PI); Larkin, ME (Co-I, SC); Meigs, J (Co-I); Chambers, B (SC, past); Dushkin, A (SC, past); Rocchio, G (SC, past); Yepes, M (SC, past); Steiner, B (RC); Dulin, H (RC, past); Cayford, M (RS); Chu, K (RS); DeManbey, A (RS); Hillard, M (RS); Martin, K (RS); Thangthaeng, N (RS); Gurry, L (RS, past); Kochis, R (RS, past); Raymond, E (RS, past); Ripley, V (RS, past); Stevens, C (RS, past)

MedStar Health Research Institute/ MedStar Baltimore: Park, J (PI); Aroda, V (PI, past); Ghazi, A (Co-PI); Magee, M (Co-I); Ressing, Ann (Co-I); Loveland, A (SC); Hamm, M (SC); Hurtado, M (SC); Kuhn, A (SC); Leger, J (SC); Manandhar, L (SC); Mwicigi, F (SC); Sanchez, O (SC); Young, T (SC)

Miami VA Healthcare System/University of Miami: Garg, R (PI), Lagari-Libhaber, V (PI); Florez, HJ (PI, past); Valencia, WM (PI, past); Marks, J (Co-PI, past); Casula, S (Co-I); Oropesa-Gonzalez, L (SC); Hue, L (SC, past); Cuadot, A (SC, past); Nieto-Martinez, R (SC, past); Riccio Veliz, AK (SC, past); Gutt, M (RC, past); Kendal, YJ (RS, past); Veciana, B. (RS, past)

Oregon Health & Science University: Ahmann, A (PI); Aby-Daniel, D (Co-I); Joarder, F (Co-I); Morimoto, V (Co-I); Sprague, C (Co-I); Yamashita, D (Co-I); Cady, N (SC); Rivera-Eschright, N (SC); Kirchhoff, P (SC, past); Morales Gomez, B (RC); Adducci, J (RC, past); Goncharova, A (RC, past)

Pacific Health Research and Education Institute/VA Pacific Islands: Hox, SH (PI); Petrovitch, H (Co-PI); Matwichyna, M (SC); Jenkins, V (SC, past); Broadwater, L (RS); Ishii, RR (RS); Bermudez, NO (RS; past)

Pennington Biomedical Research Centre: Hsia, DS (PI); Cefalu, WT (PI, past); Greenway, FL (Co-I); Waguespack, C (Co-I); King, E (SC); Fry, G (SC); Dragg, A (SC); Gildersleeve, B (SC); Arceneaux, J (SC); Haynes, N (SC, past); Thomassie, A (SC, past); Pavlionis, M (SC, past); Bourgeois, B (RC, past); Hazlett, C (RS)

San Diego VA Medical Centre: Mudaliar, S (PI); Henry, R (PI, past); Boeder, S (Co-I, past); Pettus, J (Co-I, past); Diaz, E (SC); Garcia-Acosta, D (SC); Maggs, S (SC); DeLue, C (SC, past); Stallings, A (SC, past); Castro, E (RC, past); Hernandez, S (RC, past)

Southwestern American Indian Centre: Krakoff, J (PI); Curtis, JM (Co-I); Killean, T (SC); Khalid, M (SC); Joshevama, E (RC, past); Diaz, E (RS); Martin, D (RS); Tsingine, K (RS); Karshner, T (RS, past)

St. Luke's-Roosevelt Hospital: Albu, J (Co-PI); Pi-Sunyer, FX (Co-PI, past); Frances, S (Co-I); Maggio, C (SC, past); Ellis, E (RC); Bastawrose, J (RC, past); Gong, X (RS)

SUNY Downstate Medical Centre/New York Hospital-Queens: Banerji, MA (PI); August, P (Co-I); Lee, M (Co-I); Lorber, D (Co-I); Brown, NM (SC, RC); Josephson, DH (SC); Thomas, LL (SC, RC); Tsovian, M (SC, RC); Cherian, A (SC, RC, past); Jacobson, MH (RS); Mishko, MM (RS)

The University of North Carolina Diabetes Care Centre: Kirkman, MS (PI); Buse, JB (Co-I); Diner, J (Co-I); Dostou, J (Co-I); Machineni, S (Co-I); Young, L (Co-I); Bergamo, K (Co-I, past); Goley, A (Co-I, past); Kerr, J (Co-I, past); Largay, JF (Co-I, past); Guarda, S (SC); Cuffee, J (SC, past); Culmer, D (SC, past); Fraser, R (RC); Almeida, H (RC, past); Coffer, S (RC, past); Debnam, E (RC, past); Kiker, L (RC, past); Morton, S (RC, past); Josey, K (RS); Fuller, G (RS, past)

University of Alabama Birmingham: Garvey, WT (PI); Cherrington, AL (Co-PI); Dyer, D (SC); Lawson, MCR (SC); Griffith, O (SC, past); Agne, A (RC); McCullars, S (RC)

University of Cincinnati/Cincinnati VA Medical Centre: Cohen, RM (PI); Craig, J (SC); Rogge, MC (SC; past); Burton, K (SC, past); Kersey, K (SC, RC, past); Wilson, C (SC, past); Lipp, S (RC, past); Vonder Meulen, MB (RC, past); Adkins, C (RS); Onadeko, T (RS)

University of Colorado-Denver/VA: Rasouli, N (PI); Baker, C (Co-I); Schroeder, E (Co-I, past); Razzaghi, M (Co-I); Lyon, C (Co-I, past); Penaloza, R (Co-I, past); Underkofler, C (SC); Lorch, R (SC); Douglass, S (SC, past); Steiner, S (SC, past)

University of Iowa: Sivitz, WI (PI); Cline, E (SC); Knosp, LK (SC); McConnell, J (SC, past); Lowe, T (RC)

University of Michigan: Herman, WH (PI); Pop-Busui, R (Co-PI); Tan, MH (Co-I); Martin, C (SC); Waltje, A (SC, RC); Katona, A (SC); Goodhall, L (SC, past); Eggleston, R (RC, past); Kuo, S (RS); Bojescu, S (RS, past); Bule, S (RS, past); Kessler, N (RS, past); LaSalle, E (RS, past); Whitley, K (RS, past)

University of Minnesota: Seaquist, ER (PI); Bantle, A (Co-I); Harindhanavudhi, T (Co-I); Kumar, A (Co-I); Redmon, B (Co-I); Bantle, J (Co-I, past); Coe, M (SC); Mech, M (SC); Taddese, A (RC); Lesne, L (RS); Smith, S (RS)

University of Nebraska Medical Centre/Omaha VA: Desouza, C (PI); Kuechenmeister, L (Co-I); Shivaswamy, V (Co-I); Burbach, S (SC); Rodriguez, MG (SC); Seipel, K (SC); Alfred, A (SC, past); Morales, AL (SC; past); Eggert, J (RS); Lord, G (RS); Taylor, W (RS, past); Tillson, R (RS, past)

University of New Mexico: Schade, DS (PI); Adolphe, A (Co-PI); Burge, M (Co-PI, past); Duran-Valdez, E (SC); Martinez, J (RC, past); Bancroft, A (RS); Kunkel, S (RS); Ali Jamaleddin Ahmad, F (RS, past); Hernandez McGinnis, D (RS, past); Pucchetti, B (RS, past); Scripsick, E (RS, past); Zamorano, A (RS, past)

UT Health San Antonio: DeFronzo, RA (PI); Cersosimo, E (Co-PI); Abdul-Ghani, M (Co-I); Triplitt, C (Co-I); Juarez, D (SC); Mullen, M (SC); Garza, RI (SC, past); Verastiqui, H (SC, past); Wright, K (RC, past); Puckett, C (RS)

University of Texas-Southwestern Medical Centre: Raskin, P (PI); Rhee, C (Co-I, past); Abraham, S (SC); Jordan, LF (SC); Sao, S (SC); Morton, L (SC, past); Smith, O (SC, past); Osornio Walker, L (RC, past); Schnurr-Breen, L (RC, past); Ayala, R (RS); Kreymer, RB (RS); Sturgess, D (RS, past)

VA Puget Sound Health Care System/University of Washington: Utzschneider, KM (PI); Kahn, SE (Co-I); Alarcon-Casas Wright, L (Co-I); Boyko, EJ (Co-I); Tsai, EC (Co-I); Trence, DL (Co-I, past); Trikudanathan, S (Co-I, past); Fattaleh, BN (SC); Montgomery, BK (SC, past); Atkinson, KM (RS); Kozedub, A (RS); Concepcion, T (RS, past); Moak, C (RS, past); Prikhodko, N (RS, past); Rhothisen, S (RS, past)

Vanderbilt University: Elasy, TA (PI); Martin, S (SC); Shackelford, L (RC, RS, past); Goidel, R (RS); Hinkle, N (RS); Lovell, C (RS); Myers, J (RS); Lipps Hogan, J (RS, past)

Washington University: McGill, JB (PI); Salam, M (Co-I); Schweiger, T (SC, RC); Kissel, S (SC, RC, past); Recklein, C (SC, past); Clifton, MJ (RS)

Yale University/Fair Haven Community Health Centre/West Haven VA Medical Centre: Tamborlane, W (PI); Camp, A (Co-I); Gulanski, B (Co-I); Inzucchi, SE (Co-I); Pham, K (Co-I); Alguard, M (SC, RC); Gatcomb, P (SC); Lessard, K (SC); Perez, M (SC); Iannone, L (RC); Magenheimer, E (RC); Montosa, A (RC)

Study Units

NIH/NIDDK (Sponsor): Cefalu, WT (Director, Division of Diabetes, Endocrinology and Metabolic Diseases); Fradkin, J (Director, Division of Diabetes, Endocrinology and Metabolic Diseases, past); Burch, HB (Project Scientist); Bremer, AA (Project Scientist, past)

Chairman's Office, Massachusetts General Hospital, Harvard Medical School: Nathan, DM (Study Chair, Study Co-PI)

Executive Committee: Nathan, DM (Study Chair, Study Co-PI); Lachin, JM (U01 Contact PI, Study Co-PI); Buse, JB (Co-I); Kahn, SE (Co-I); Krause-Steinrauf, H (Co-I, Project Director); Larkin, ME (Co-I, SC); Tiktin, M (Co-I, SC); Wexler, D (PI); Burch, HB (Program Scientist); Bremer, AA (Project Scientist, past)

Coordinating Centre, The George Washington University Biostatistics Centre: Lachin, JM (U01 Contact PI, Study Co-PI); Krause-Steinrauf, H (Co-I, Project Director, Co-PI EDS Substudy); Younes, N (Co-I); Bebu, I (RS); Butera, N (RS); Fagan, A (RS); Gao, Y (RS); Ghosh, A (RS); Gramzinski, MR (RS); Hall, SD (RS); Kazemi, E (RS); Legowski, E (RS); Suratt, C (RS); Tripputi, M (RS); Arey, A (RS, past); Backman, M (RS, past); Bethepu, J (RS, past); Buys, CJ (RS, past); Liu, H (RS, past); Lund, C (RS, past); Mangat Dhaliwal, P (RS, past); McGee, P (RS, past); Mesimer, E (RS, past); Ngo, L (RS, past)

Central Biochemical Laboratory, University of Minnesota Advanced Research and Diagnostic Laboratory: Steffes, M (PI); Seegmiller, J (Co-I); Saenger, A (Co-I, past); Arends, V (SC); Gabrielson, D (SC, past)

Drug Distribution Centre, VA Cooperative Studies Program Clinical Research Pharmacy Coordinating Centre: Conner, T (PI); Warren, S (PI, past); Day, J (RS); Huminik, J (RS); Scrymgeour, A (RS)

ECG Reading Centre, EPICARE, Wake Forest University: Soliman, EZ (PI); Pokharel, Y (PI, past), Zhang, ZM (Co-I, past); Campbell, C (SC); Hu, J (SC); Keasler, L (SC); Hensley, S (SC, past); Li, Y (RS)

Economic Evaluation and Assessment Centre:

University of Michigan: Herman, WH (PI); Kuo, S (RS); Martin, C (SC); Waltje, A (SC, RC); Mihalcea, R (RS); Min, DJ (RS); Perez-Rosas, V (RS); Prosser, L (RS); Resnicow, K (RS); Ye, W (RS)

Centres for Disease Control and Prevention: Shao, H (RS); Zhang, P (RS)

Neurocognitive Coordinating Centre, Columbia University Medical Centre: Luchsinger, J (PI); Sanchez, D (SC); Assuras, S (RS)

QWB Reading Centre, University of California San Diego Health Services Research Centre: Groessl, E (PI); Sakha, F (SC); Chong, H (SC, past); Hillery, N (RS)

Collaborators

Cardiovascular Adjudication Advisor: Everett, BM (Brigham and Women's Hospital)

Collaborating Investigators (Recruitment Sites): Abdouch, I (University of Nebraska Medical Centre/Omaha VA); Bahtiyar, G (SUNY Downstate Medical Centre); Brantley, P (Pennington Biomedical Research Centre (LSU)); Broyles, FE (Swedish Medical Centre); Canaris, G (University of Nebraska Medical Centre/Omaha VA); Copeland, P (Massachusetts General Hospital); Craine, JJ (UW Valley Medical Centre); Fein, WL (Swedish Medical Centre); Gliwa, A (SUNY Downstate Medical Centre); Hope, L (SUNY Downstate Medical Centre); Lee, MS (SUNY Downstate Medical Centre); Meiners, R (Pennington Biomedical Research Centre (LSU)); Meiners, V (Pennington Biomedical Research Centre (LSU)); O'Neal, H (Pennington Biomedical Research Centre (LSU)); Park, JE (UW Valley Medical Centre); Sacerdote, A (SUNY Downstate Medical Centre; Sledge, Jr., E (Pennington Biomedical Research Centre (LSU)); Soni, L (SUNY Downstate Medical Centre); Steppel-Reznik, J (Massachusetts General Hospital); Turchin, A (Massachusetts General Hospital)

Beta Cell Ancillary Study: Brooks-Worrell, B (University of Washington); Hampe, CS (University of Washington); Palmer, JP (University of Washington); Shojaie, A (University of Washington)

Continuous Glucose Monitoring Sub-study: Higgins, J (Massachusetts General Hospital; Harvard Medical School)

Emotional Distress Sub-study: Golden, S (Johns Hopkins University); Gonzalez, J (Yeshiva University; Albert Einstein College of Medicine); Naik, A (Baylor College of Medicine); Walker, E (Albert Einstein College of Medicine)

National Diabetes Education Program (NDEP) Sub-study: Doner Lotenberg, L (Hager Sharp); Gallivan, JM (National Institutes of Health); Lim, J (Hager Sharp); Tuncer, DM (National Institutes of Health)

Recruitment Ancillary Study: Behringer-Massera, S (The Mount Sinai Hospital, Beth Israel Medical Centre)

ESM Figure 1. Overview of missing values for the different variables included in the analyses.

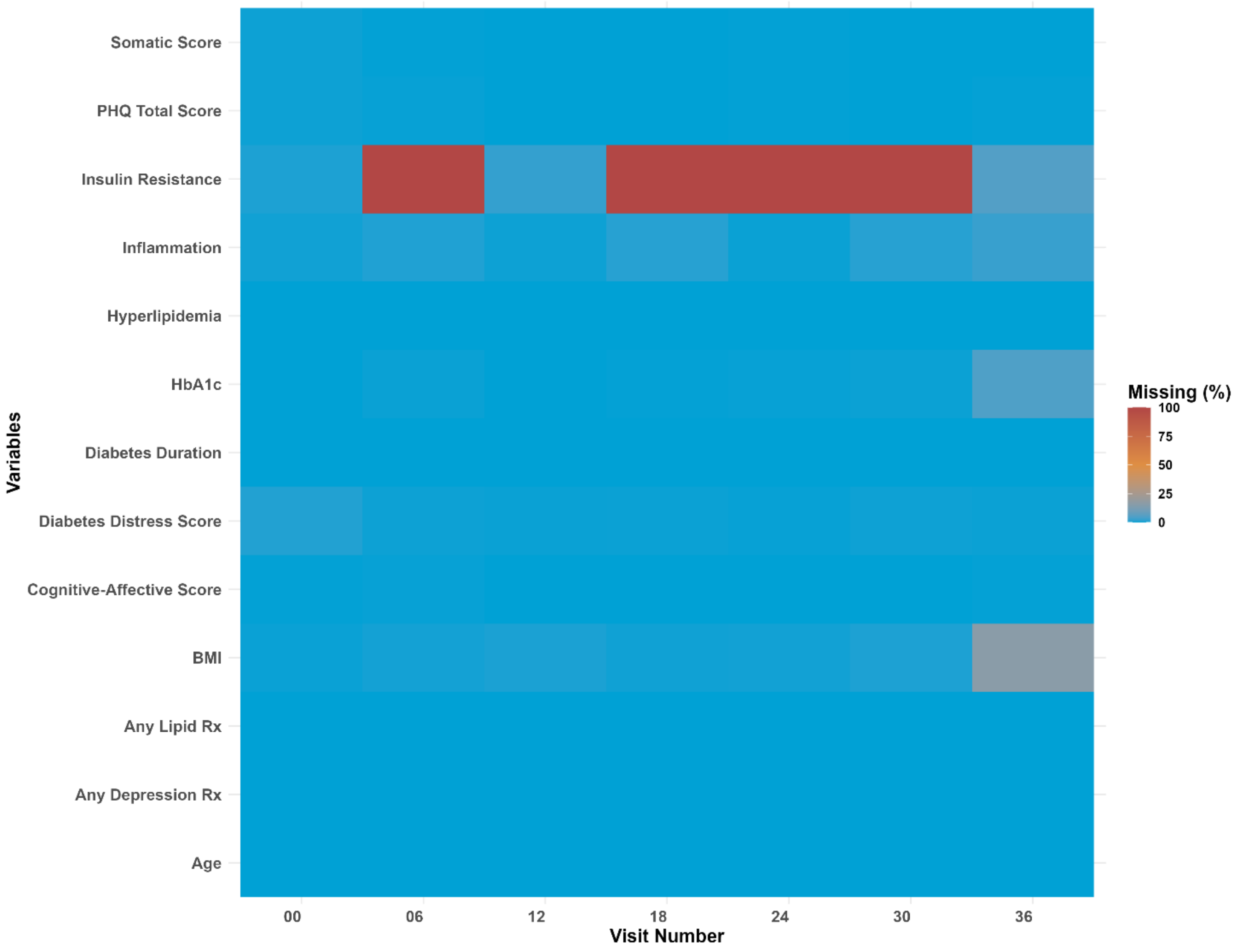

ESM Figure 2. Relationship between PHQ-8 total score and insulin resistance ($\log_e$ HOMA-IR) at baseline.

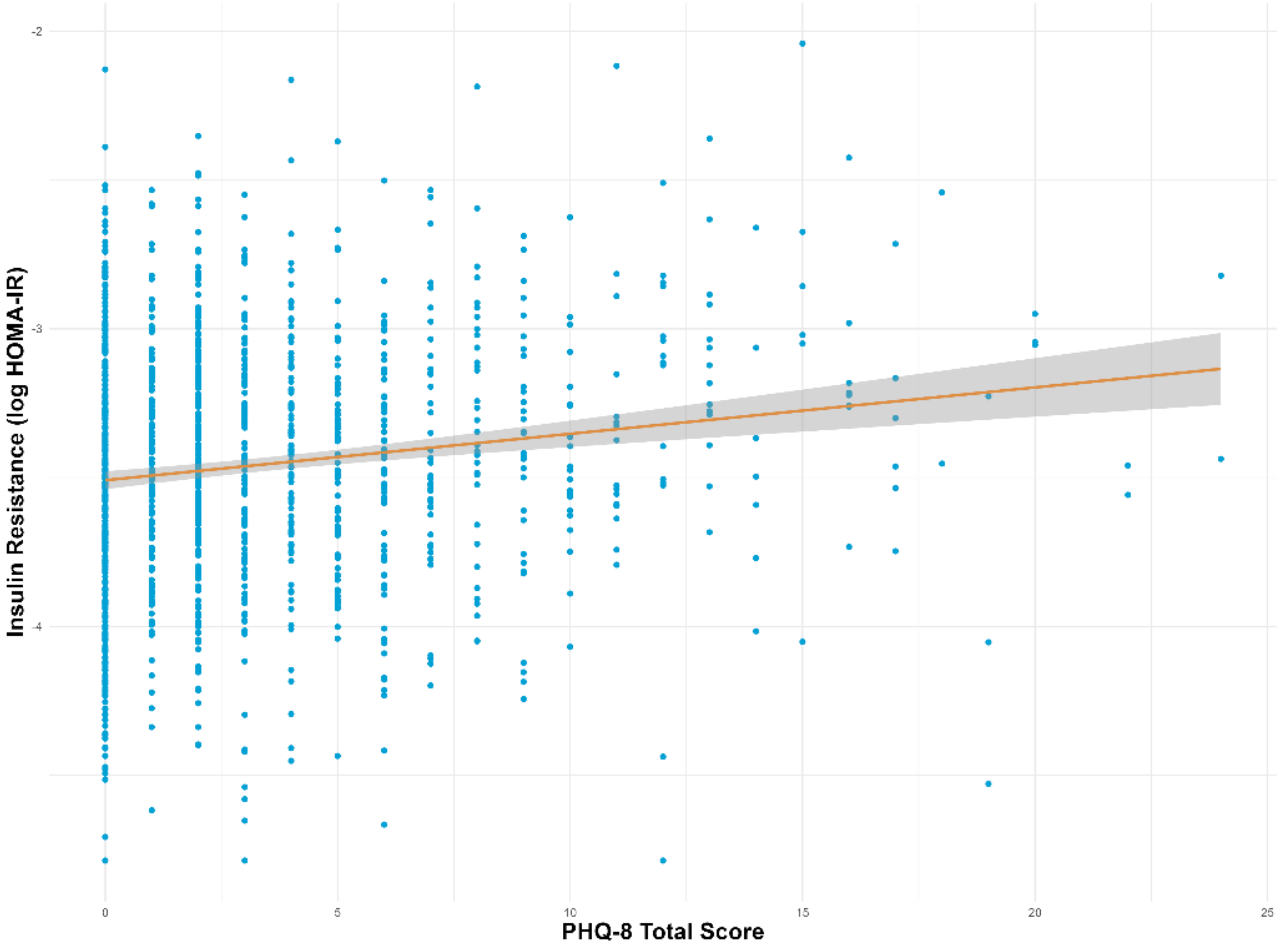

ESM Figure 3. Relationship between PHQ-8 total score and insulin resistance (log_e HOMA-IR) at baseline with Locally Estimated Scatterplot Smoothing (LOESS)

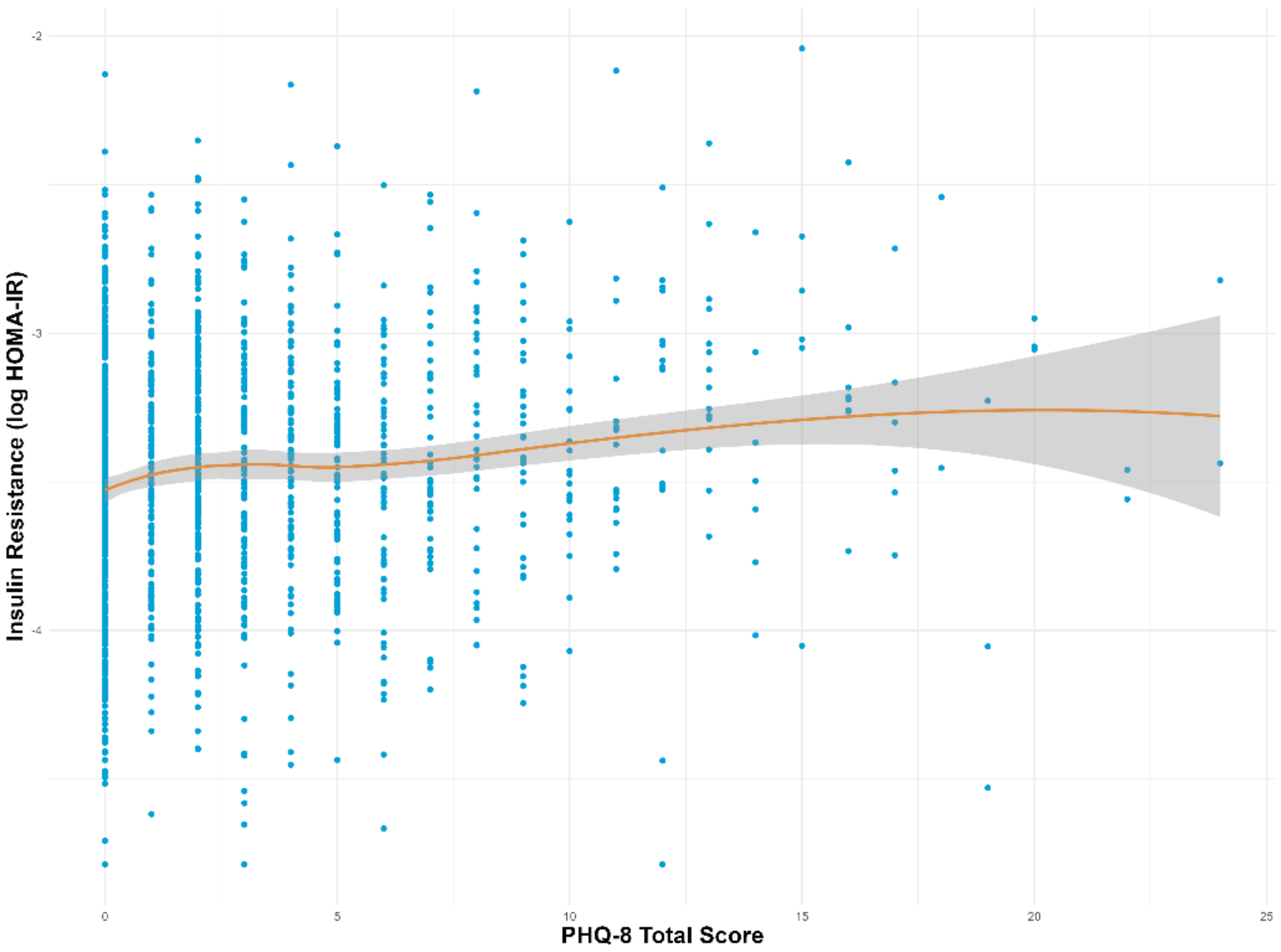

ESM Figure 4. Relationship between PHQ-8 total score and inflammation ($\log_e$ hsCRP) at baseline.

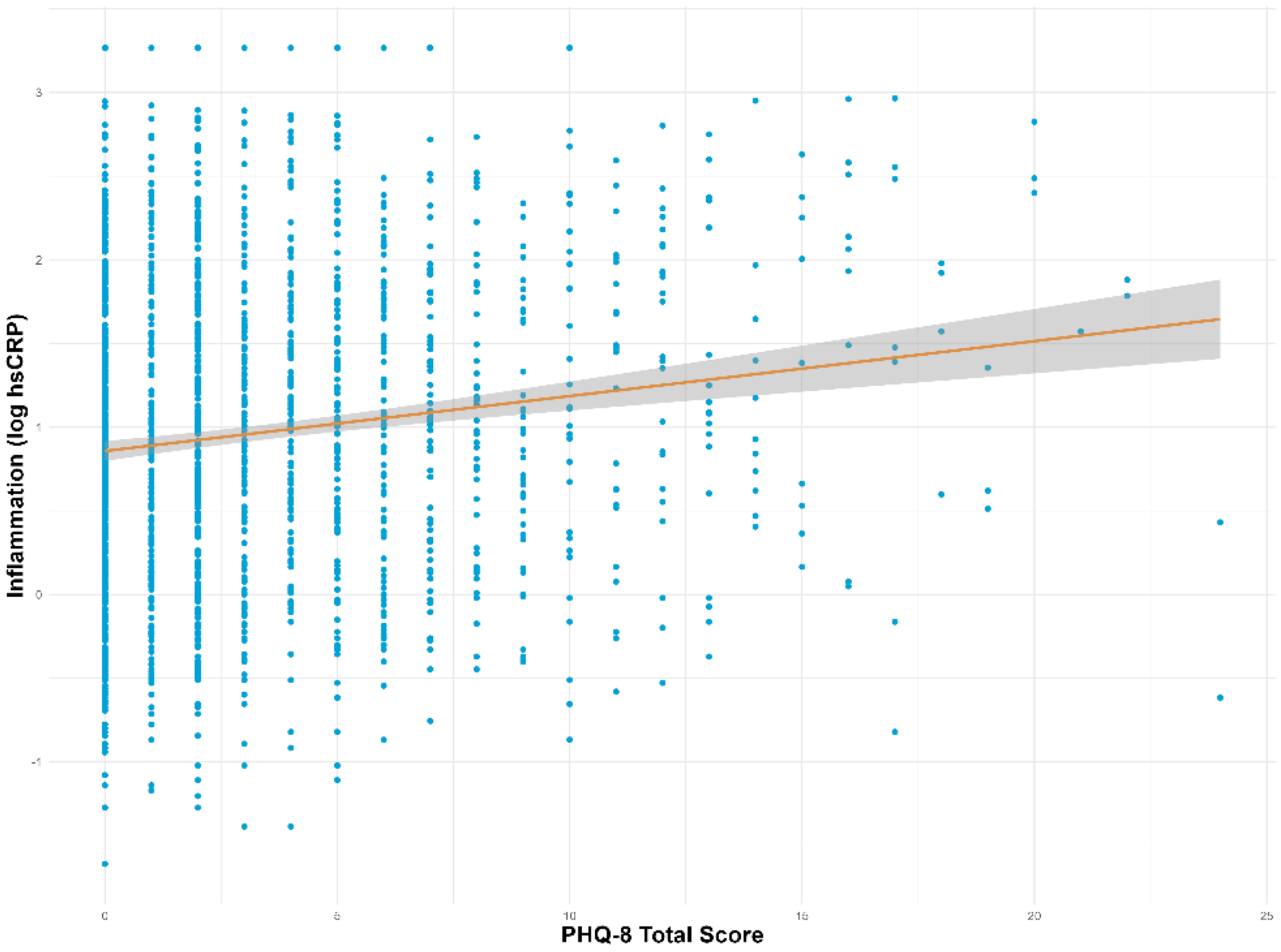

ESM Figure 5. Relationship between PHQ-8 total score and inflammation (log_e hsCRP) at baseline with Locally Estimated Scatterplot Smoothing (LOESS)

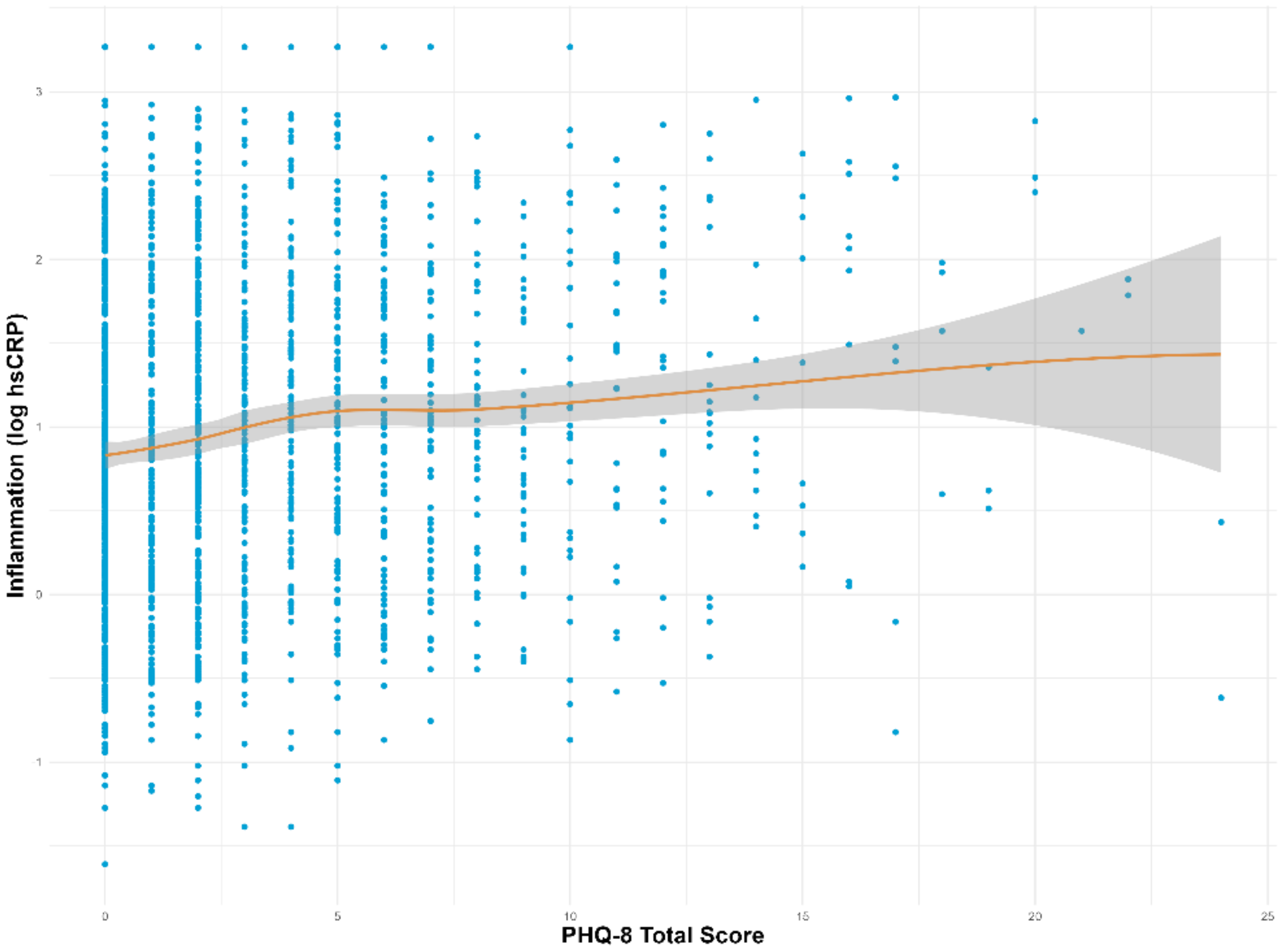

ESM Table 1. Sensitivity analysis for the associations with insulin resistance excluding those taking insulin after randomisation

**Models**	**Independent Variable**	**Insulin Resistance (log$_e$ HOMA-IR, *N* = 1321)**			
		**Estimates**	**SE**	**95% CI**	***p*-value**
Overall Depressive Symptoms					
**Adjusted**[a]	PHQ-8 Total Score	-0.015	0.001	-0.003, -0.002	0.787
**Unadjusted**	PHQ-8 Total Score	0.017	0.003	0.011, 0.022	**<0.001**
Depressive Symptoms Scores					
**Adjusted**[a]	Somatic Symptom	-0.004	0.010	-0.022, 0.015	0.692
	Cognitive-Affective Symptom	0.0001	0.010	-0.019, 0.020	0.993
**Unadjusted**	Somatic	0.126	0.021	0.085, 0.167	**<0.001**
	Cognitive-Affective	0.085	0.022	0.041, 0.129	**<0.001**

[a] Adjusted for sex, race/ethnicity, randomized treatment group, and baseline measurements of: age, duration of diabetes, HbA1c, BMI, taking antidepressants, taking lipid-lowering medications, lipid profiles, levels of inflammation and insulin resistance, and diabetes distress. Models accounted for the within participant correlation over time using a random effect. Participants were excluded when they took rescue insulin at any given study visit (*n*=276/1321).

ESM Table 2. Sensitivity analysis for the cross-sectional associations with insulin resistance (comparing Matsuda Index & HOMA-IR)

Models	Independent Variable	Insulin Resistance (Matsuda Index, *N* = 1184)				Insulin Resistance ($\log_e$ HOMA-IR, *N* = 1184)			
		**Estimates**	**SE**	**CI**	***p*-value**	**Estimates**	**SE**	**CI**	***p*-value**
Overall depressive symptoms									
**Adjusted**[a]	PHQ-8 Total Score	-0.008	0.004	-0.017, 0.000	0.060	0.006	0.003	0.001, 0.012	**0.028**
**Unadjusted**	PHQ-8 Total Score	-0.026	0.005	-0.036, -0.017	**<0.001**	0.019	0.003	0.012, 0.025	**<0.001**
Depressive symptoms scores									
**Adjusted**[a]	Somatic	-0.081	0.031	-0.143, -0.019	**0.010**	0.054	0.021	0.013, 0.095	**0.010**
	Cognitive-Affective	-0.006	0.033	-0.070, 0.058	0.855	0.021	0.022	-0.022, 0.064	0.334
**Unadjusted**	Somatic	-0.212	0.034	-0.279, -0.146	**<0.001**	0.148	0.024	0.101, 0.194	**<0.001**
	Cognitive-Affective	-0.111	0.036	-0.182, -0.040	**0.002**	0.085	0.025	0.035, 0.135	**<0.001**

[a] Adjusted for baseline diabetes distress, duration of type 2 diabetes, age, sex, race/ethnicity, randomized treatment group, BMI, HbA1c, taking lipid-lowering drugs, lipid profiles, and taking antidepressants. Models accounted for the within-participant correlation over time using a random effect.

ESM Table 3. Sensitivity analysis for the longitudinal associations with insulin resistance (comparing Matsuda & HOMA-IR)

**Models**	**Independent Variable**	**Insulin Resistance (Matsuda Index, *N* = 1284)**				**Insulin Resistance ($\log_e$ HOMA-IR, *N* = 1284)**			
		**Estimates**	**SE**	**CI**	***p*-value**	**Estimates**	**SE**	**CI**	***p*-value**
Overall depressive symptoms									
**Adjusted**[a]	PHQ-8 Total Score	-0.002	0.003	-0.007, 0.003	0.335	-0.00001	0.001	-0.003, 0.003	0.992
**Unadjusted**	PHQ-8 Total Score	-0.022	0.004	-0.030, -0.014	**<0.001**	0.016	0.003	0.011, 0.022	**<0.001**
Depressive symptoms scores									
**Adjusted**[a]	Somatic	-0.023	0.018	-0.059, 0.013	0.211	-0.003	0.011	-0.023, 0.018	0.808
	Cognitive-Affective	-0.004	0.019	-0.042, 0.033	0.817	0.004	0.011	-0.017, 0.026	0.687
**Unadjusted**	Somatic	-0.175	0.031	-0.235, -0.115	**<0.001**	0.123	0.022	0.080, 0.165	**<0.001**
	Cognitive-Affective	-0.095	0.033	-0.159, -0.031	**0.004**	0.085	0.023	0.040, 0.130	**<0.001**

[a] Adjusted for baseline levels of insulin resistance (Matsuda and HOMA-IR, respectively), baseline diabetes distress, baseline duration of type 2 diabetes, age, sex, race/ethnicity, randomized treatment group, BMI, HbA1c, taking lipid-lowering drugs, lipid profiles, taking antidepressants at baseline. Models accounted for the within-participant correlation over time using a random effect.

ESM Table 4. Association between each depression symptom at baseline with inflammation and insulin resistance at baseline

Models	Independent Variable	Inflammation (log_e hsCRP, *N* = 1739)				Insulin Resistance (log_e HOMA-IR, *N* = 1321)			
		**Estimates**	**SE**	**CI**	***p*-value**	**Estimates**	**SE**	**CI**	***p*-value**
**Adjusted[a]**	Little interest or pleasure in doing things	0.045	0.031	-0.015, 0.105	0.143	0.026	0.016	-0.005, 0.056	0.098
	Feeling down, depressed, irritable or hopeless	-0.020	0.035	-0.089, 0.050	0.579	0.024	0.018	-0.011, 0.060	0.185
	Trouble falling or staying asleep, or sleeping too much	0.020	0.021	-0.021, 0.062	0.333	0.016	0.011	-0.005, 0.037	0.132
	Feeling tired or having little energy	0.017	0.024	-0.029, 0.063	0.464	0.036	0.012	0.013, 0.059	**0.002**
	Poor appetite or overeating	0.050	0.026	-0.001, 0.101	0.055	0.035	0.013	0.009, 0.061	**0.009**
	Feeling bad about yourself – or that you are a failure or have let yourself or your family down	-0.002	0.035	-0.070, 0.066	0.957	0.020	0.018	-0.014, 0.055	0.251
	Trouble concentrating on things, such as school work, reading or watching television	0.030	0.032	-0.032, 0.092	0.347	0.010	0.016	-0.021, 0.040	0.538
	Moving or speaking so slowly that other people could have noticed? Or the opposite – being so fidgety or restless that you have been moving around a lot more than usual	-0.043	0.045	-0.131, 0.045	0.341	0.052	0.022	0.008, 0.095	0.021
**Unadjusted**	Little interest or pleasure in doing things	0.139	0.035	0.069, 0.208	**<0.001**	0.060	0.018	0.024, 0.096	**0.001**
	Feeling down, depressed, irritable or hopeless	0.121	0.041	0.041, 0.200	**0.003**	0.065	0.021	0.023, 0.106	**0.002**
	Trouble falling or staying asleep, or sleeping too much	0.068	0.025	0.020, 0.117	**0.006**	0.044	0.013	0.019, 0.069	**<0.001**
	Feeling tired or having little energy	0.139	0.027	0.086, 0.192	**<0.001**	0.089	0.014	0.062, 0.116	**<0.001**
	Poor appetite or overeating	0.197	0.029	0.140, 0.254	**<0.001**	0.103	0.015	0.074, 0.132	**<0.001**
	Feeling bad about yourself – or that you are a failure or have let yourself or your family down	0.116	0.041	0.037, 0.196	**0.004**	0.074	0.021	0.032, 0.115	**<0.001**
	Trouble concentrating on things, such as school work, reading or watching television	0.098	0.037	0.026, 0.170	**0.008**	0.048	0.018	0.012, 0.084	**0.010**

Moving or speaking so slowly that other people could have noticed? Or the opposite – being so fidgety or restless that you have been moving around a lot more than usual	0.031	0.053	-0.074, 0.135	0.567	0.074	0.027	0.022, 0.127	**0.006**

[a] Adjusted for diabetes distress, Body-Mass Index, taking lipid-lowering drugs, lipid profiles, taking antidepressants, sex, age, race/ethnicity.

ESM Table 5. Associations between each depression symptom at baseline and longitudinal inflammation and insulin resistance.

Models	Independent Variable	Inflammation (log_e hsCRP, N = 1739)				Insulin Resistance (log_e HOMA-IR, N = 1321)			
		**Estimates**	**SE**	**CI**	***p*-value**	**Estimates**	**SE**	**CI**	***p*-value**
**Adjusted[a]**	Little interest or pleasure in doing things	0.028	0.017	-0.005, 0.061	0.102	0.002	0.008	-0.013, 0.018	0.788
	Feeling down, depressed, irritable or hopeless	0.032	0.019	-0.007, 0.070	0.105	0.003	0.009	-0.016, 0.021	0.788
	Trouble falling or staying asleep, or sleeping too much	0.009	0.012	-0.013, 0.032	0.426	0.001	0.005	-0.010, 0.011	0.913
	Feeling tired or having little energy	0.019	0.013	-0.007, 0.044	0.151	-0.006	0.006	-0.018, 0.006	0.341
	Poor appetite or overeating	0.035	0.014	0.007, 0.062	0.015	-0.004	0.007	-0.017, 0.010	0.596
	Feeling bad about yourself – or that you are a failure or have let yourself or your family down	0.028	0.019	-0.009, 0.066	0.136	-0.004	0.009	-0.022, 0.014	0.653
	Trouble concentrating on things, such as school work, reading or watching television	0.032	0.017	-0.002, 0.066	0.068	0.001	0.008	-0.014, 0.017	0.865
	Moving or speaking so slowly that other people could have noticed? Or the opposite – being so fidgety or restless that you have been moving around a lot more than usual	0.049	0.024	0.001, 0.096	0.048	-0.025	0.011	-0.047, -0.003	0.028
**Unadjusted**	Little interest or pleasure in doing things	0.152	0.034	0.086, 0.218	**<0.001**	0.054	0.017	0.020, 0.087	**0.002**
	Feeling down, depressed, irritable or hopeless	0.153	0.038	0.078, 0.229	**<0.001**	0.060	0.019	0.022, 0.098	**0.002**
	Trouble falling or staying asleep, or sleeping too much	0.071	0.023	0.026, 0.117	**0.002**	0.039	0.012	0.016, 0.062	**<0.001**
	Feeling tired or having little energy	0.154	0.025	0.104, 0.204	**<0.001**	0.071	0.013	0.046, 0.096	**<0.001**
	Poor appetite or overeating	0.210	0.027	0.156, 0.263	**<0.001**	0.087	0.014	0.059, 0.114	**<0.001**
	Feeling bad about yourself – or that you are a failure or have let yourself or your family down	0.141	0.038	0.066, 0.216	**<0.001**	0.061	0.020	0.023, 0.099	**0.002**
	Trouble concentrating on things, such as school work, reading or watching television	0.116	0.035	0.048, 0.184	**<0.001**	0.043	0.017	0.010, 0.076	**0.011**

Moving or speaking so slowly that other people could have noticed? Or the opposite – being so fidgety or restless that you have been moving around a lot more than usual	0.082	0.050	-0.017, 0.181	0.105	0.039	0.025	-0.010, 0.087	0.120

[a] Adjusted for baseline levels of inflammation and insulin resistance, respectively, baseline diabetes distress, duration of type 2 diabetes, age, sex, race/ethnicity, randomized treatment group, BMI, HbA1c, taking lipid-lowering drugs, lipid profiles, taking antidepressants, also accounted for the within-participant correlation over time using a random effect.
